# Whole transcriptome analysis of the silicon response of the diatom *Thalassiosira pseudonana*

**DOI:** 10.1186/1471-2164-13-499

**Published:** 2012-09-20

**Authors:** Roshan Prakash Shrestha, Benoit Tesson, Trina Norden-Krichmar, Stephen Federowicz, Mark Hildebrand, Andrew E Allen

**Affiliations:** 1 Scripps Institution of Oceanography, University of California, San Diego, California, 92037, USA; 2 J. Craig Venter Institute, San Diego, California, 92121, USA; 3 Systems Biology Research Group, University of California, San Diego, California, 92093, USA

**Keywords:** Cell cycle, Cell wall, Diatom, Microarray, Silicon efflux, Silicon metabolism, Silicon transporter, Synchrony, *Thalassiosira pseudonana*, Transcriptomics

## Abstract

**Background:**

Silicon plays important biological roles, but the mechanisms of cellular responses to silicon are poorly understood. We report the first analysis of cell cycle arrest and recovery from silicon starvation in the diatom *Thalassiosira pseudonana* using whole genome microarrays.

**Results:**

Three known responses to silicon were examined, 1) silicified cell wall synthesis, 2) recovery from silicon starvation, and 3) co-regulation with silicon transporter (SIT) genes. In terms of diatom cell wall formation, thus far only cell surface proteins and proteins tightly associated with silica have been characterized. Our analysis has identified new genes potentially involved in silica formation, and other genes potentially involved in signaling, trafficking, protein degradation, glycosylation and transport, which provides a larger-scale picture of the processes involved. During silicon starvation, an overrepresentation of transcription and translation related genes were up-regulated, indicating that *T. pseudonana* is poised to rapidly recover from silicon starvation and resume cell cycle progression upon silicon replenishment. This is in contrast to other types of limitation, and provides the first molecular data explaining the well-established environmental response of diatoms to grow as blooms and to out-compete other classes of microalgae for growth. Comparison of our data with a previous diatom cell cycle analysis indicates that assignment of the cell cycle specific stage of particular cyclins and cyclin dependent kinases should be re-evaluated. Finally, genes co-varying in expression with the SITs enabled identification of a new class of diatom-specific proteins containing a unique domain, and a putative silicon efflux protein.

**Conclusions:**

Analysis of the *T. pseudonana* microarray data has provided a wealth of new genes to investigate previously uncharacterized cellular phenomenon related to silicon metabolism, silicon’s interaction with cellular components, and environmental responses to silicon.

## Background

Silicon plays important roles in biology, but at the molecular level, very little is known about how cellular components recognize, interact with, and process this element. By silicon, in dissolved form, we mean silicic acid [Si(OH)_4_, and in solid form, silica [SiO_2_. Silicon’s biological roles include essentiality in the formation of bones and connective tissue in vertebrates [1,2], a beneficial, and possibly essential role in plants for optimal productivity and increased disease resistance [3], and as a structural material in the cell walls of plants and single-celled protists [3,4]. The most abundant silicifying organisms on the planet are unicellular microalgae called diatoms, which make silica-based cell walls, and which can readily deplete their environment for trace amounts of silicon. Diatoms have demonstrated cellular responses to the depletion or re-addition of silicon. For most diatoms, under silicon depletion the cell cycle arrests and under subsequent silicon replenishment, the cell cycle progresses, cellular growth and division processes are stimulated, and new cell walls are synthesized [5]. From the available data [6], silicon is not tightly tied into other aspects of cellular metabolism, although given the paucity of appropriate studies, this should be considered with caution. However, based on our current understanding, it is clear that the three major cellular processes affected by or involving silicon are the cell cycle, silicon transport, and cell wall synthesis.

Mechanisms triggering cell cycle arrest or progression upon silicon starvation or addition remain unknown, but probably involve signaling processes that result in control over cell cycle-related genes. Additionally, it has been suggested that the ecological success of diatoms is due to their ability to recover quickly from silicon starvation, which allows them to outcompete other species during blooms [7]. How diatoms can rapidly recover and resume the cell cycle and growth after a starvation period remains unclear. Nearly all diatoms exhibit a dependence on silicon for cell cycle progression, and limitation for silicon will arrest the cell cycle at particular stages, which can vary depending on the species [8]. Oftentimes, the majority of cells in a culture arrest at the same stage, and upon silicon replenishment, they progress synchronously through the cell cycle, which enables characterization of cell cycle-related processes [5]. Synchronization allows for enrichment of genes and proteins induced during these processes, enabling their identification and characterization [9].

A molecular interaction between silicon and diatom cellular components has been shown to occur during silicon transport. Silicic acid at low concentrations enters into the diatom cell via silicic acid transporters (SITs) which specifically recognize and transport silicic acid across lipid bilayer membranes [10,11]. At higher concentrations, the small uncharged silicic acid molecule can diffuse across membranes [12]. The SITs were the first proteins shown to specifically interact with soluble silicon and not cause its precipitation [10], therefore they are models for understanding how other proteins may interact with silicon. The demonstration that the SITs interact with silicon opens the possibility that enzymes may also be able to do so, however to date, this has not been demonstrated. Some data suggested a direct effect of silicon on enzyme activity, specifically, DNA polymerase [13], but subsequent work using purified enzyme failed to demonstrate a direct effect on activity [14]. This suggests that the induction of DNA polymerase activity was a secondary effect stemming from induction of cell cycle progression after silicon replenishment. To date, no enzyme has been shown to have altered activity directly resulting from the presence or absence of silicon, although diatom carbonic anhydrases have been shown to use silica as a buffering agent [15].

**Figure 1:**
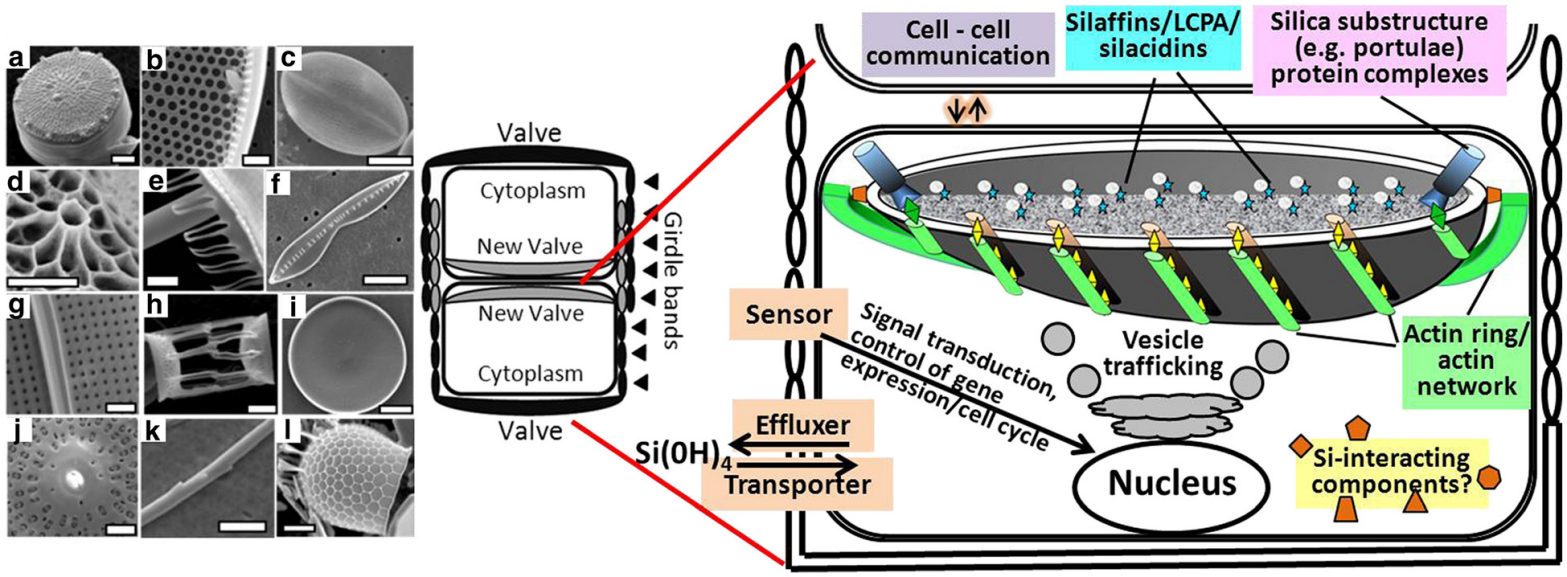
** Diatom cell wall structures and components.** Left, SEMs showing the diversity of diatom valve structures from different species. Scale bars **a, k)** 1 μm, **b)** 5 μm **c, f)** 10 μm **d)** 500 nm **e, g, h, j, l)** 2 μm, **i)** 50 μm. Center, diagram of a diatom cell during valve formation. Right, diagram of a dividing diatom cell highlighting major aspects of silicon’s interaction with the cell or valve formation (colored boxes) that are major focus areas of this study. Specific components are described in detail in the text.

Silicon’s role is better defined in terms of diatom cell wall synthesis. The diatom cell wall, called the frustule, is made of silica in a vast variety of species-specific shapes and structures (Figure 1) on the nanometer to micrometer size scale [4,16]. Cell wall formation occurs intracellularly in the silica deposition vesicle (SDV), in which the two major structural components of the wall, the valves and the girdle bands (Figure 1), are made. After completion, these structures are exocytosed by an unknown mechanism [17] to form portions of the new cell wall. The intracellular origin of the SDV is poorly understood, and could involve both secretory and endocytotic processes [18], however specific components involved in trafficking are uncharacterized. Transport of silicification precursors into the SDV is also completely uncharacterized. The protein machinery involved in membrane dynamics for cell division including cytokinesis, SDV formation, and exocytosis remain unknown. 

We have some understanding of silica structure formation from analysis of the organic components associated with the cell wall silica or SDV. After extensive cleaning of organic material from cell walls, followed by dissolution of the silica, two classes of proteins, the silaffins and silacidins, and long chain polyamines (LCPAs), were isolated (Figure 1). These are apparently directly involved in the nanoscale silica polymerization process [19], but do not assemble the silica into the higher order structures characteristic of the cell wall. Proteins associated with the girdle bands called cingulins characterized in *T. pseudonana* formed structures indicative of higher order organization [20]. It was suggested that chitin fibrils were involved in formation of the *T. pseudonana* valve [21], which could relate to higher order structure formation. Insoluble organic matrices with silica polymerization activity were also described in the valves of other diatom species [20]. Microtubule and microfilament networks are tightly associated with the SDV, and observations suggest that microtubules are involved in its positioning and strengthening, and actin microfilaments are involved in the mesoscale patterning of silica, and microscale structure formation by defining the leading edge of the SDV [22,23]. Actin and microtubules must assemble outside the SDV, and yet apparently influence the organization of components in the SDV lumen, which has been proposed to occur via SDV membrane-associated proteins that bridge the extra- and intra- components [24,25]. Given the complexity of diatom silica structures on the nano- and meso-scale [4,16] other unknown SDV-associated components are likely to be involved in the formation of substructures such as nanopores and large pores in the cell wall called portulae (Figure 1). A rigorous characterization of the SDV proteome has not been possible, due to the inability to isolate a pure SDV fraction. An alternative way to characterize SDV components is to identify genes up-regulated during cell wall formation.

We developed a synchronized culture procedure for *T. pseudonana*, based on recovery from silicon limitation that enabled identification of a distinctive cell cycle-specific transcriptional response for cell wall associated proteins such as the silaffins [5,9]. This approach should be amenable to study other cellular responses to silicon. Two previous analyses of diatom whole transcriptome expression in response to silicon availability were performed [26,27]. One study was done on *Phaeodactylum tricornutum*, a diatom that does not require silicon for growth, and in several morphotypes, lacks a silicified wall [28]. The goal of that study was to investigate non-cell cycle related silicon processes, and cell wall synthesis was not evaluated. Thirteen genes were up-regulated under silicon-free medium and 210 were up-regulated in silicon-replete medium [26]. The other study, performed on *T. pseudonana*, monitored transcript changes resulting from growth under two different silicon concentrations, including one in which growth was limited, but not arrested [27]. This study identified 159 genes up-regulated under the lower silicon condition, which were suggested to be involved in silica formation processes [27]. However, under silicon-limited conditions in *T. pseudonana* cell division is decreased or ceases [5], thus up-regulation of silica formation related genes is unlikely. Supporting this concept is the fact that a gene previously shown to be diagnostic of cell wall synthesis, silaffin 3 [9], was not present in this dataset [27]. Although the genes in this study [27] may have relevance for growth under low silicon conditions, they do not encompass a silicon starvation or cell cycle arrest response.

The synchrony approach developed for *T. pseudonana* should allow evaluation of whole transcriptome responses for various silicon-related cellular processes. One process to be studied is cell wall synthesis, which has not been subject to a whole transcriptome analysis. Monitoring transcript changes may be especially valuable considering that many diatom cell wall synthesis genes are unlikely to have homologs in other organisms, and similarity to a diagnostic gene expression pattern may be the only approach to identifying them. The synchrony approach should allow evaluation of the silicon starvation response, which may provide insight into general aspects of cellular silicon metabolism, and how diatoms recover quickly from limitation. Nutrient starvation and replenishment commonly induces changes in expression of genes involved in metabolizing the limited nutrient [29], and thus other genes involved in silicon metabolism may be identified, as well as the basis of silicon-limited cell cycle arrest. Finally, because the SITs are definitively involved in silicon metabolism and responsible for silicon’s presence in the cell, they could serve as markers to identify other co-regulated genes that may also be involved in silicon transport or metabolism.

Here we use Affymetrix microarrays to evaluate the whole genome mRNA expression profile during synchronized cell cycle progression in *T. pseudonana*. We identified 485 genes significantly up-regulated during the period of valve formation, which are potentially involved in different aspects of the cell division/cell wall synthesis process. An additional 533 genes were significantly up-regulated during silicon starvation and thus, are potentially involved in the control of cell cycle progression in response to silicon availability. Twenty four genes were identified that exhibited co-regulation with SIT1 and SIT2. Analysis of the data has provided a wealth of new genes to investigate previously uncharacterized cellular phenomenon related to silicon metabolism, silicon’s interaction with cellular components, and environmental responses to silicon.

## Results and Discussion

### Generation of datasets

The overall dataset contained 11,756 unique genes from the *T. pseudonana* genome database, version 3.0. Validation of the microarray data was performed on a biological replicate using qRT-PCR ( Additional file 1). The Affymetrix data was statistically analyzed (p-value cut off of <0.05) and 7440 genes with at least at one time point significantly different than 0 h were selected. To identify genes potentially involved in cell wall formation, we selected genes with a similar expression profile as Tpsil3 by comparing the hybridization signal at 7 and 8 h with 0 and 4 h. The 2 hr timepoint was not considered because expression at this time could be related to either girdle band synthesis or the small percentage of cells that are arrested in G2/M at this time point [5]. We identified 485 genes significantly (T-test; P < 0.05) up-regulated at 7 or 8 h, during which valve formation takes place, and termed the silaffin-like response genes (SLRG) dataset ( Additional file 2). To identify genes potentially involved in the silicon starvation response, we selected genes significantly up-regulated (P < 0.05) at 0 h (533 total) compared to 2, 4, 7, 8, 9 h. These were called silicon starvation response genes (SSRG) dataset ( Additional file 3). Genes up-regulated at timepoints other than 0 h were not analyzed because of potential complications in interpretation due to multiple cellular processes occurring. A third dataset identified 24 genes co-regulated with SIT1 and 2, which had similar patterns compared with each other. SIT3 expression did not substantially vary during the synchrony; therefore cluster analysis was not performed on it.

### Silaffin-like response genes (SLRG)

#### Initial analysis of the SLRG dataset

**Figure 2:**
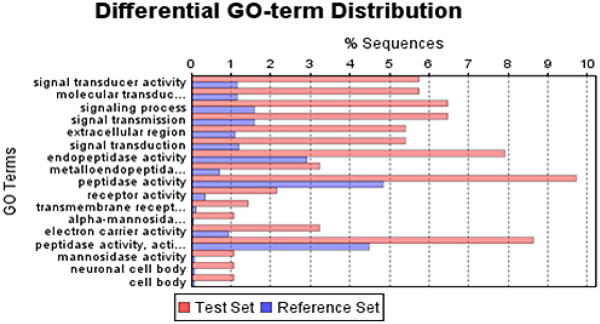
** Bar diagram of functional category enrichment analysis (Fisher's Exact Test) of SLRGs (Test Set) and total data set (Reference Set).** Y-axis represents significant enrichment of GO terms and X-axis shows the relative frequency of the term.

We functionally categorized the SLRG subset based on generic GO slim analysis ( Additional file 4) according to molecular function, biological process, and cellular compartment. GO categories that are overrepresented in this dataset may contain genes of interest for cell wall synthesis. We performed Fisher’s Exact test (FDR cutoff of p < 0.005) using the Blast2GO module FatiGO [30] (Figure 2) and identified substantially over represented genes in the SLRG subset, which included proteins involved in signaling processes/signal transduction, peptidase/metalo-endopeptidases, extracellular proteins, and mannosidase activities (Figure 2). The SLRG dataset contained 175 genes specific to diatoms (*T. pseudonana,* and *P. tricornutum*) ( Additional file 5). 

#### Proteins involved in signal transduction

Signal transduction components are over-represented in the SLRG dataset (Figure 2; Additional file 2) which might be explained by the need of a tight control over timing of events and communication between the two daughter cells during division (Figure 1). Two phospholipase C genes were up-regulated at 7 and 8 h (Thaps3_262103 and 38652). Phospholipase C is responsible for the cleavage of PIP2 into diacylglycerol (DAG) and IP3, and IP3 binds to specific receptors leading to the release of calcium in the cytoplasm. Together, DAG and calcium activate protein kinase C, which controls the activity of other proteins by phosphorylation. Phosphorylation during diatom cell wall synthesis has been previously documented [31]. Other signal transduction components in the SLRG dataset were three G protein coupled receptors (Thaps3_22601, 7839 and 7830), two adenylate guanylate cyclases (Thaps3_262719, 263505), 3 cAMP dependant protein kinases (Thaps3_33798, 5802, 22399), and one cAMP specific phosphodiesterase (Thaps3_262976). G protein coupled receptors activate adenylate cyclase for the release of cAMP, which activates cAMP dependent kinases that phosphorylate other proteins. Phosphodiesterases degrade cAMP and regulate the intracellular cAMP gradient. In diatoms [32] levels of cAMP and cGMP increase prior to cell division and decrease before cells separate. G protein coupled receptors can also activate Phospholipase C. Three Elongation Growth Factor (EGF) domain containing proteins were also present; EGF is usually found in the extracellular domain of membrane bound or secreted proteins. One protein (Thaps3_7122) was a homolog of Sig2 (a sexually induced protein) from *Thalassiosira weissflogii*[33], and two (Thaps3_22281, 24894) share homology with each other and with proteins from the NOTCH family, which are transmembrane receptors important for cell-cell communication. Binding of the ligand to the NOTCH receptor is involved in the control of gene expression and control of cell fate [34].

Thaps3_7839 shares homology with a GABAb receptor, which is activated by ligand binding, indicative of a response to extracellular stimuli. In diatoms, two daughter cells are formed within a mother cell (Figure 1), and only subsequently does valve synthesis and exocytosis occur, which must be coordinately completed to enable separation of the two daughter cells. Such coordination could require the two daughter cells to communicate, which could involve transduction of signals through G protein coupled receptors and the cAMP signaling pathway. NOTCH proteins are triggered by cell to cell contact, and in our dataset the NOTCH homologs were specifically up-regulated during valve formation – consistent with a close interaction between daughter cells (Figure 1). Other signal sensors were identified, including two histidine kinases (Thaps3_262720, 262298), and a protein sharing homology with aureochrome - a blue light photoreceptor/transcription factor regulating morphogenesis in stramenopiles [35].

Three isoforms of calcium-dependent protein kinases (Thaps3_14030, 14378, 36648) containing calcium binding EF hand motifs were also found in the SLRG dataset. Isoforms of Ca-binding protein kinases participate in multiple distinct signaling pathways from defense to plant growth and development [36-39]. These genes were up-regulated at 7 and 8 h only; thus, their specific up-regulation only during valve formation suggests a role in this process.

These data are consistent with the occurrence of a trigger for signal transduction-induced protein phosphorylation during cell wall formation. The overabundance of signal transduction components suggests a new aspect of cell wall synthesis control.

#### Proteins involved in protein degradation

Proteins involved in protein degradation were also overrepresented in the SLRG subset (Figure 2; Additional file 2), with 8 proteins related to the ubiquitination pathway (Thaps3_267964, 2575, 21152, 16926, 268040, 33603, 33696) and three proteins as components of the proteasome complex (Thaps3_34018, 22996, 264206). Additionally, four proteinase/peptidase proteins were present as well as two proteinase inhibitors. The ubiquitin related proteins included a cullin, a hydrophobic protein providing a scaffold for ubiquitin ligase. Protein localization data suggested that ubiquitin was involved in the degradation of proteins mediating the formation of pores inside the valve of *Navicula pelliculosa*[40]. Valve formation likely involves extensive re-organization of components, and these data suggest that protein degradation could play a significant role. Additionally proteins involved in silicification processes like silacidins and silaffins are known to undergo proteolytic cleavage [41,42].

#### Extracellular proteins

Seven proteins sharing sequence homology with previously characterized copper induced proteins were present in the SLRG dataset (Thaps3_12594, 20786, 269653, 9432, 20795, 1822-bd, 12594) ( Additional file 2). Most of these were not previously suggested to be associated with cell wall processes, however one (p150), which possesses a chitin binding domain, is associated with the girdle bands and was suggested to be involved in the stabilization of the cell during division [43]. Two of the other proteins have chitin binding domains and 3 possess an allergen V5/Tpx-1-related domain. A total of 11 proteins with the allergen V5/Tpx-1-related domain are in the SLRG dataset, compared with 15 in the entire genome, hence this domain is likely involved in cell wall-associated processes.

*T. pseudonana* is known to secrete chitin fibrils through the valve structures called portulae [44,45]. Chitin has also been proposed to be involved in valve formation [21,46]. The *T. pseudonana* genome contains more than 20 genes encoding chitinases and chitin-binding proteins [46]. We found 8 of these proteins in the SLRG dataset, among them, two chitinase genes and six genes encoding proteins with chitin-binding domains. In general, chitin synthases and chitinases function in coordination to build chitin fibers [47]. However none of the chitin synthase genes are significantly up-regulated during valve synthesis, so their role in valve formation remains unclear.

#### Glycosylation related proteins

Silaffins are highly glycosylated and sulfated, and contain mannose, galactose, glucose and glucuronic acid [48]. Substituted mannans are components of the diatom cell wall, however their role and localization remain unclear. Chiovitti et al. [49] suggested that mannans and other neutral sugars are present in the silica matrix in different diatom species including *T. pseudonana* and could play a role in the silicification process. We observed that α-mannosidases were overrepresented in the SLRG subset. Thaps3_34968 and 40232 were expressed similarly to Tpsil3, with significant up-regulation during girdle band and valve formation, whereas Thaps3_263310 was up-regulated only during valve formation ( Additional file 2). Mannosidases are important in the process of N-glycan synthesis but could also be involved in the maturation of mannans associated with the cell wall. In addition, we found three glycosyltransferases, two alpha mannosyltransferases, involved in the transfer of mannosyl residues on dolichol phosphate in the N-glycosylation pathway and one N-acetylglucosaminyltransferase which is involved in the formation of heparin sulfate during glycosaminoglycan synthesis.

#### Vesicle trafficking related proteins

Cell wall synthesis requires vesicle trafficking and fusion (Figure 1) – the SDV is apparently built from the fusion of small vesicles that are most likely Golgi-derived [17,50]. One homolog of SNARE (Thaps3_262803), involved in vesicle trafficking from the ER to the Golgi, and one homolog of NSF protein (Thaps3_269412), involved in membrane fusion between vesicles and cellular compartments, were present in the SLRG dataset ( Additional file 2). Cell division protein 48 (CDC48, Thaps3_267952) was also up-regulated, CDC48 is an ATPase required for protein degradation and membrane fusion that seems to be involved in the budding and transfer of membrane from the ER to the Golgi. This protein was previously identified in a proteomics study as being possibly involved in diatom cell wall synthesis [9]. ARP1 (Thaps3_269504) and one cytoplasmic dynein (Thaps3_269188) were also induced at 7 and 8 h. ARP1 is part of the dynactin complex which is involved in the microtubule-dependent transport of vesicles through the cytoplasm and requires dynein as a motor protein. Additionally, we found one gene with homology to sorting nexin 1 (Thaps3_262863), which is a component of the retromer complex (recycling of transmembrane proteins from endosome to the TGN) - it contains a phox homology domain which has affinity for phosphoinositide and a Cterm BAR domain. The Cterm BAR domain mediates formation of a banana-shaped dimer which has affinity to curved membranes and also induces membrane deformation [51]. Subunit B from the clathrin complex (Thaps3_26212) was up-regulated - the clathrin complex is known to be involved in endocytic processes, which may play a role in SDV formation [18]. These genes are excellent candidates to be the first described molecular components involved in SDV trafficking, and their further characterization should enable an understanding of SDV assembly, as well as provide specific markers for this unique organelle.

#### Transporters

Much of the functional capability of an organelle is related to its transporters, which define what metabolites and compounds can cross its membrane. In the SLRG dataset, Thaps3_262743 is a choline transporter ( Additional file 2). The expression patterns were similar to that of Tpsil3 throughout the synchronized cell cycle, suggestive of a role in silicification ( Additional file 6). Choline is an important component of osmoregulation, which may be a critical factor in the SDV, which accumulates a solid material during silica deposition. Seven genes (Thaps3_5607, 22820, 932, 21480, 20595, 22830, and 22844) that encode multiple membrane-spanning proteins of unknown function with a conserved domain were also identified. Among them Thaps3_20595, 22820, 22830 and 22844 were up-regulated at 7 and 8 h ( Additional file 2). Although the proteins showed some similarity to PF04515 (choline transporter, PFAM), which is a member of the superfamily cl04558 (Choline_transpo super family, SUPERFAMILIES), functional characterization of orthologs does not support a role in choline transport [52]. The presence of several copies of these genes up-regulated during valve formation is consistent with an important yet unknown role in SDV function.

We also detected four sugar transporters, including polyol transporters Thaps3_14028 and Thaps3_37974. A sugar efflux transporter/bidirectional sugar transporter Thaps3_30921 and a nucleotide-sugar transporter/UDP-galactose translocator Thaps3_16344 were also identified. It is possible that these sugar transporters are involved in silicification processes, however, we have not detected any endoplasmic reticulum (ER) signal peptides in these proteins. Polyols such as glycerol, sorbitol, and mannitols could serve as osmolytes during silicification inside the SDV, and/or they can serve as a protein stabilizer during silicification [53].

#### Additional genes identified

Synthesis of the SDV membrane will require a substantial amount of phospholipids, and several proteins from the membrane phospholipid biosynthesis pathway were present in the SLRG dataset ( Additional file 2). Many chloroplast related genes were present in the dataset, including light harvesting proteins and proteins involved in pigment synthesis ( Additional file 2). Chloroplast related genes up-regulated during cell division were also identified in a synchronized culture of a pennate diatom [54]. We also found many proteins involved in the response to oxidative stress (thioredoxin, peroxidase). Several glycolytic enzymes were also identified. One cyclophilin containing a signal peptide was up-regulated, this enzyme is responsible for the isomerisation of peptidic bonds from trans to cis and plays a role as immunosuppressant, protein chaperone and modulator of protein function and expression [55]. 2, 3-*cis*-3, 4-*trans*-3, 4-dihydroxy-L-proline has been identified as a major component of the diatom cell wall [56], thus this cyclophilin gene could be involved in the processing of cell wall proteins.

#### Genes involved in cell wall synthesis or production of precursors identified by homology

**Figure 3:**
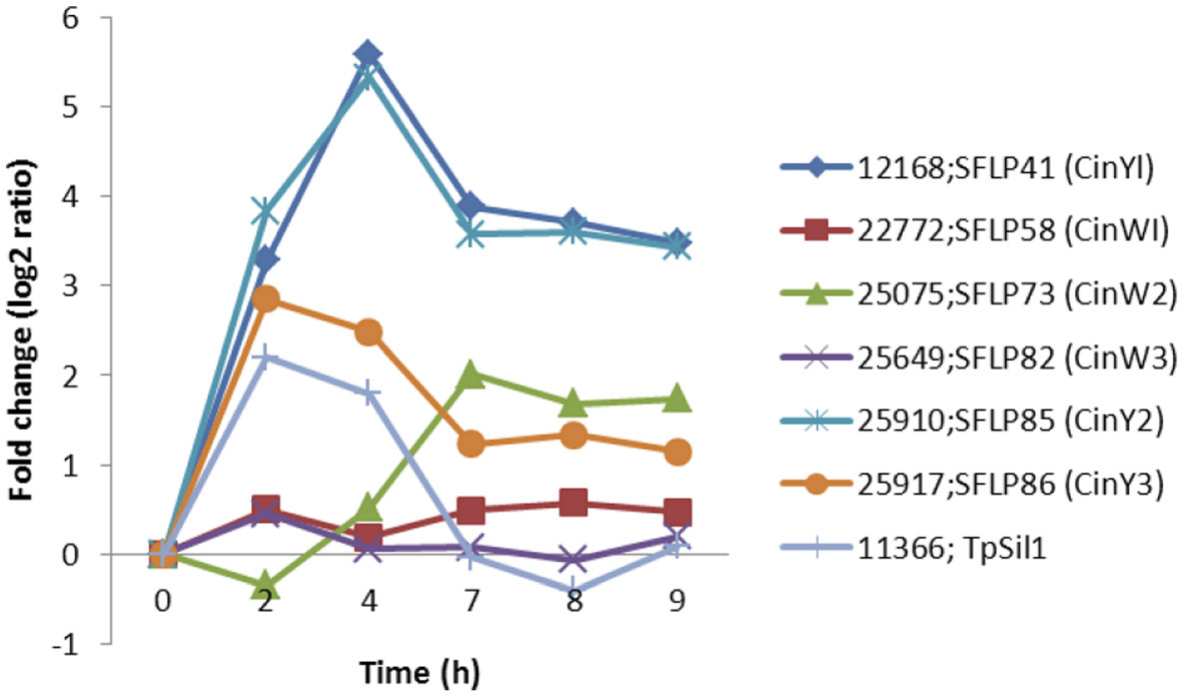
** Expression pattern of girdle-band associated cingulins compared with Tpsil1.** Two cingulins W1 and W2 were present in SLRG dataset.

Two girdle band associated proteins, the cingulins W1 and W2 [20] were present in the SLRG dataset. CinW1 was moderately up-regulated at the 2, 7, 8 and 9 hour time points, corresponding to the time of girdle band (2 h) and valve synthesis (7–8 h), and CinW2 was highly up-regulated at 7 hr, decreased slightly and remained constant at 8 and 9 h (Figure 3). Localization experiments indicated that these two cingulins were in girdle bands close to the valves [20]; perhaps they play roles in valve formation by mediating the attachment of the valve with girdle bands. CinW3 (not in the SLRG dataset) was slightly up-regulated at 2 hr, and then levels decreased (Figure 3). All three CinY proteins were highly up-regulated at 2 and 4 h and then expression decreased but remained high during valve formation. Up-regulation during S-phase (4 h), which is not typically associated with cell wall synthesis processes, may suggest other roles for cingulins. The expression profiles of CinYs were highly similar to Tpsil1 (Thaps3_11366), suggesting that both sets of proteins may be involved in girdle band synthesis or early processes of cell wall formation. The expression profiles of CinY and CinW were different suggesting a distinct role of these protein subfamilies. 

#### Identification of novel genes that might be involved in silicification process

In the study by Mock et al. [27], 159 genes were up-regulated under their silicon limitation conditions, with 84 stimulated by both Si and iron limitation and 75 exclusively by Si. These genes were suggested to be involved in silicification bioprocesses, however there are two considerations in this regard. The first is that in the Mock et al. study, culture density was still increasing at the time of harvesting [27], indicating that true silicon limitation may not have occurred. Indeed the authors measured 6 μmol· L^-1^ silicon in the growth medium at harvesting. The second consideration is that under true silicon limitation, the majority of *T. pseudonana* cells become arrested in the G1 phase of the cell cycle, and several hours must pass after silicon replenishment before new valve synthesis occurs [5], which is when the majority of genes involved in silicification are expected to be up-regulated. Out of the 159 genes identified in the Mock et al. study [27], only 14 genes were present in the SLRG subset ( Additional file 7).This confirms the differences in cellular status resulting from different culture conditions in the two studies (which used the same strain of *T. pseudonana*), and suggests that the genes identified by Mock et al. [27] are not likely to be related to silicification.

Proteins associated with the SDV are expected to have an ER signal peptide. A complicating factor is that diatom gene models are frequently truncated at the amino-terminal end, which is the location of the signal peptide. Thus bioinformatically-based evaluation of proteins with ER or SDV targeting generally will provide an underestimate of the true number. Regardless, among the SLRG, 74 genes possess an ER signal peptide, 26 of these are unknown and have no defined domains, 32 are predicted to contain at least 1 transmembrane domain, including 13 unknown that are potential SDV membrane associated proteins. The remaining 13 unknown ER signal peptide containing soluble proteins are potential intralumenal SDV components. These genes represent primary target for the identification of new components involved in silicification process in diatoms. Identification of the role of these unknown genes will require investigation using genetic manipulation approaches. Localization of these proteins using proteins fused to GFP will be a first step for the identification of SDV associated proteins. Sequence characteristics of some of these proteins are described below.

#### Cluster analysis of genes following the TpSil3 pattern

Diatom silica precipitating proteins lack sequence homology to each other and to other proteins, thus their identification is problematic. We applied a hierarchical cluster analysis with TpSil3 using Genesis v1.7.6 software [57] on the SLRG data to identify similarly regulated genes that might also be involved in silica formation.

**Figure 4:**
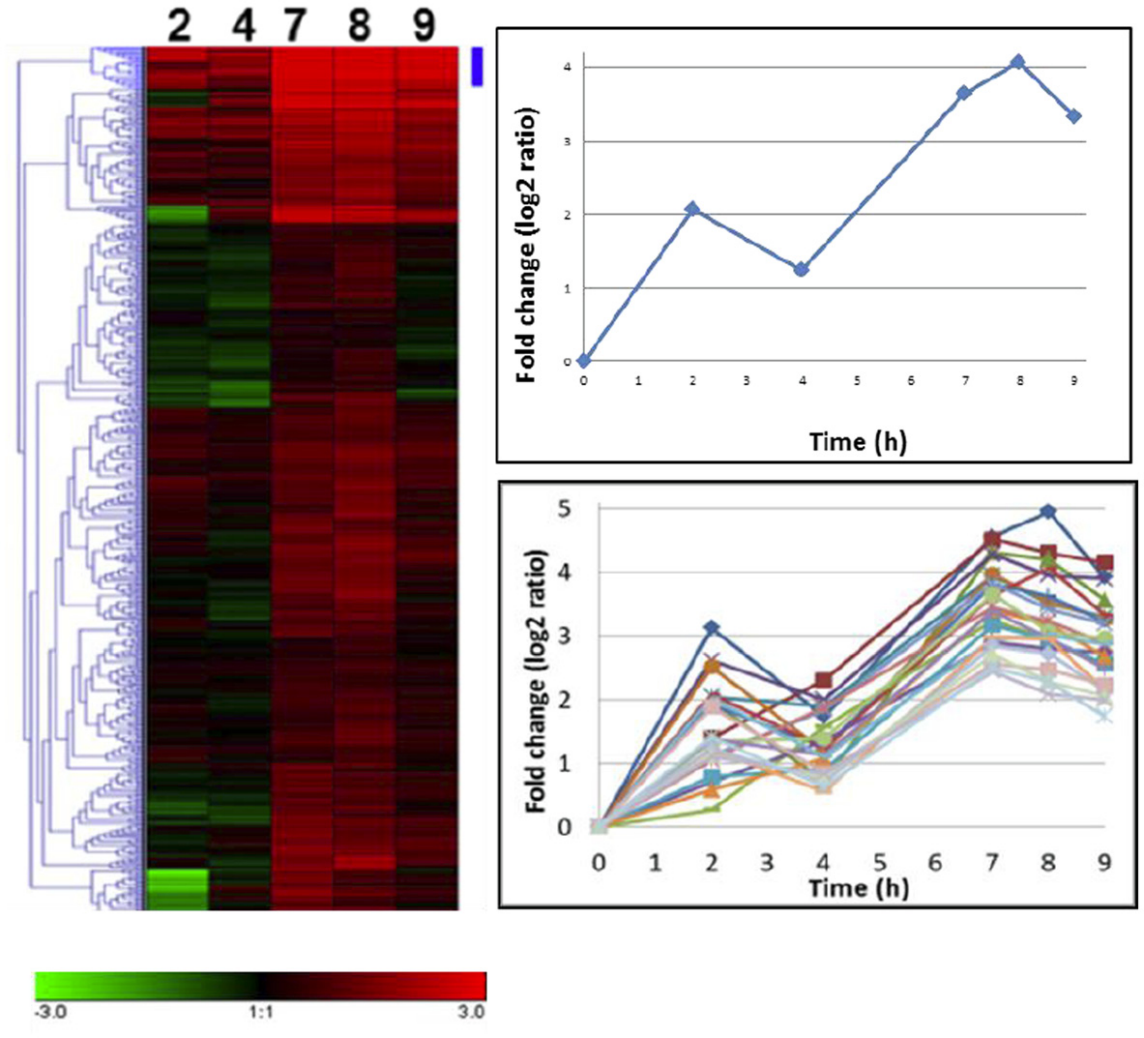
** Hierarchical clustered expression profile of 485 silaffin-like response genes (SLRGs), and specific responses of the Tpsil3 cluster.** Left) Heatmap of the SLRG data. Columns correspond to log2ratio (fold change) of the time points (2, 4, 7, 8 and 9 h) relative to 0 h of a synchronized cell culture. The intensities of the colors indicate the magnitude of up-regulation (red) and down regulation (green). Black indicates no change. The Tpsil3 cluster is indicated by the blue bar. Right) upper is the expression profile of Tpsil3 and lower are the expression profiles of genes in the Tpsil3 cluster showing expression level fold changes.

The Tpsil3 gene defines the heatmap cluster with highest fold change at 7 and 8 h (Figure 4), and which contains 23 genes ( Additional file 8). Most of the cluster members have no significant similarity to other genes. After performing a Gene Ontology (GO) analysis [58], we determined that most proteins were soluble and predicted to be either secreted or to contain extracellular domains ( Additional file 8). Two recently proposed silaffin-like genes [Thaps3_24597 and 25838 - [20] in addition to Tpsil3 are present in the silaffin cluster. Silicification proteins are generally enriched in serine and lysine, on which basis Scheffel et al. [20] used a bioinformatics strategy using amino acid composition (≥ 18% serine and ≥ 10% lysine residues per 100 amino acids) and the presence of an ER signal peptide to identify 89 genes potentially involved in frustule formation. Altogether there are 11 common genes between the Scheffel and SLRG datasets, with three (including Tpsil3) in the silaffin cluster. Most of the proteins of the silaffin cluster are rich in acidic amino acids ( Additional file 8), which is common in other proteins involved in biomineralization [19,59,60]. A few proteins including Tpsil3 have predicted basic pI values, but due to extensive posttranslational modifications, the actual pI of silaffin is 3.0 [48]. 

**Figure 5:**
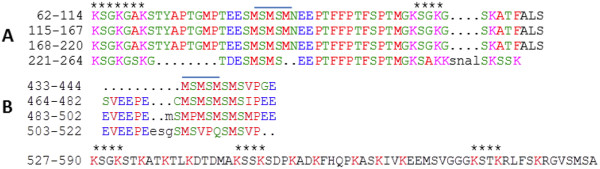
** Partial amino acid sequences of two genes of the silaffin cluster.A**) Thaps3_24597 has three complete 53 aa repeats and one partial repeat. The sequence was verified by sequencing cDNA and one extra complete repeat than predicted in the Thaps vers. 3 genome database was found. **B**) Thaps3_ 25828 C-terminal sequence has three repeats and KXXK motifs. KXXK motifs are denoted by asterisks. MSMSM motifs are overlined.

Repeated amino acid sequences are characteristics of the silaffins and silacidin [41,61], therefore we analyzed the clustered proteins using RADAR (Rapid Automatic Detection and Alignment of Repeats in protein sequences) software to detect if any repeats were present [62]. These were identified in two genes (Figure 5). Thaps3_24597 is 322 amino-acids long and contains three 53 a repeats with 18.72% serine and 12.81% lysine and several KXXK peptide motifs, mostly KSSK (Figure 5), which are known to be involved in precipitation of silica and formation of silica nanoparticles [63]. Thaps3_25838 had an ER signal peptide and also contained short imperfect repeats, but KXXK motifs were present only at the non-repeating 3’end of the protein (Figure 5). The full length protein (590 aa) contained 11.53% serine and 3.56% lysine, but the repeat stretch (residue 433–590) had 21.48% Serine and 11.11% lysine. One interesting similarity between Thaps3_25838 and 24597 is an MSMSM motif, which could have a yet to be determined common function. 

#### Cell cycle related proteins

Three cyclins and one cyclin dependent kinase are present in the SLRG dataset. Recall that *T. pseudonana* is in the G2 + M cell cycle stage under the conditions from which the SLRG dataset was generated. One protein (Thaps3_11150), which has low similarity with cyclin but contains a cyclin N-terminal domain, was highly up-regulated at 7 and 8 h, and is not described in a recent study [64]. Thaps3_11150 shares low similarity with dsCyc 6 (E-value 1E-9) a diatom specific cyclin described in *P. tricornutum* and shown to be up-regulated in G1 and S phase [64]. Another cyclin (Thaps3_11138) shares low homology with Cyclin A and is highly up-regulated at 2, 7 and 8 h, following the silaffin 3 expression profile. This protein is classified as a diatom specific cyclin by Huysman et al. [64] and shares some similarity with dsCyc7 (E-value 2E-18), which has been shown to be up-regulated in G1, and following phosphate addition after a starvation period. Another cyclin (Thaps3_ 264631) shares some homology with cyclin Y and is slightly up-regulated at 7 and 8 h, and is characterized as a U/P cyclin by Huysman et al. [64]. Thaps_264631 shares similarity with CycP1 (E-value 6E-73) from *P. tricornutum*, where it is up-regulated in G1/S. Finally one cyclin dependant kinase (Thaps3_32468) is up-regulated at 2, 7 and 8 h and absent from ref [64]. Thaps3_32468 shares similarity with hCDK5 from *P. tricornutum* (E-value 6E-173) where it was up-regulated in G1/S. There are substantial differences between the Huysman study and ours, specifically with regard to several cyclins and cyclin kinases being described as up-regulated at G1/S by Huysman et al. [64] which are up-regulated at G2 + M in our study. There are several factors that could be responsible for the differences. One is that the method of synchronization was different; in *P. tricornutum* synchronization was achieved by prolonged darkness whereas for *T. pseudonana* silicon starvation was used. In the Huysman study, the experiment was terminated before completion of the G2/M phase, meaning that a direct comparison between cell wall synthesis processes cannot be done. The diatom specific cyclins were proposed to be involved in the control of cell cycle by environmental conditions [64], however the specifics of this are not yet understood. The different physiology and silicon dependence of *P. tricornutum* and *T. pseudonana* and the low conservation between certain genes might also contribute to the differences observed. One outcome of this comparison is that more cross-species and different conditions comparative analyses should be done before assigning particular cyclins and cyclin-dependent kinases to particular stages of the cell cycle.

### Silicon starvation responsive genes (SSRG)

#### Preliminary analysis

We identified 533 genes significantly up-regulated under silicon starvation compared to subsequent time points after addition of silicate to the medium ( Additional file 3; Additional file 9). Among those genes, 151 are unknown and specific to diatoms including 82 found in *P. tricornutum*, and 69 found only in *T. pseudonana* ( Additional file 10).

Only 6 and 3 genes from the SSRG list are found to be differentially regulated in the Mock and Sapriel studies, respectively [26,27] ( Additional file 11). In the case of the Sapriel study it is not very surprising because *P. tricornutum* does not require silicon to grow and the absence of silicic acid in the medium does not arrest the cell cycle. Regarding the Mock study, which was done on the same strain of *T. pseudonana*, cells were under conditions of silicon limitation but not starvation, which means that they were still dividing, but slowly, so no cell cycle arrest was involved.

**Figure 6:**
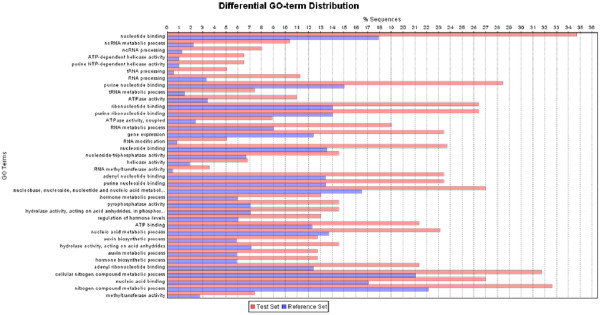
** Bar diagram of functional category enrichment analysis of SSRGs (Test Set) and total data set (Reference Set).** Y-axis represents significant enrichment of GO terms and X-axis shows the relative frequency of the term.

To explore which GO functional categories were over-represented in the SSRG subset in relation to the total dataset, we performed Fisher’s Exact test (FDR cutoff of p < 0.005) using Blast2GO (Figure 6). Substantially over represented genes in the SSRG subset were proteins involved in RNA and nucleotide processing, gene expression, hormone metabolism, nitrogen metabolism and lipid transport (Figure 6). We found parallels between the SSRGs and classes of genes identified in the phosphate starvation response of plants with the exception of protein synthesis genes, which are down-regulated in plants [29,65]. 

#### SSRG genes involved in expression regulation and cell cycle arrest

Especially overrepresented in the SSRG dataset are transcription factors and helicases which are known to bind to DNA and RNA and control transcription and translation. Two silent information regulator protein 2 (SIR2) genes are also up-regulated, SIR2 is a histone deacetylase which controls chromatin packing and inhibits transcription under conditions of phosphate starvation in plants [66]. We also found one Small Ubiquitin related Modifier (SUMO) E3 ligase, which is also up-regulated during phosphate starvation in higher plants [67], and has been proposed to regulate the activity of a transcription factor involved in the phosphate starvation response [67]. Several eukaryotic initiation and elongation factor were up-regulated in the SSRG dataset, including eIF2 and 3 and EF1, EFG and EFTu. These proteins are involved in the initiation and elongation during translation and require for protein synthesis. Additionally GCN2, a serine kinase is also up-regulated. GCN2 has been shown to phosphorylate EIF2, inducing G1 arrest in response to various stresses [68-70].

Several genes involved in the control of cell cycle progression were up-regulated, including one diatom specific cyclin (Thaps3_3215). Thaps3_3215 shares similarity with dSCyc 8 from *P. tricornutum* (E-value 5eE-29) which was found to be up-regulated at the end of the G1 phase [64]. Some genes specifically required for S phase entry include cdc123 and RIO1 [71,72]. Expression of cdc 123 protein is triggered by nutrient availability and regulates the abundance of eIF2 required for S phase entry [71]. RIO1 is an atypical protein kinase and has been shown to be involved in ribosome biogenesis [72,73]. Several ubiquitin related genes are also up-regulated, protein degradation is known to be an important phenomenon for the transition to a new cell cycle phase [74]. How diatoms sense the absence of silicic acid is unknown; an unsubstantiated possibility is that SIT3 could function as a sensor, but regardless, a signal has to be sent to trigger the changes in gene expression documented in the SSRG dataset. Different signal transduction related genes were up-regulated during silicon starvation, including two multi-sensor multi-kinases, two Ca2+/calmodulin protein kinases and several Ser/thr protein kinases.

#### Ecological relevance of SSRGs

It is well known that diatoms recover faster from silicon starvation than from nitrogen starvation [7]. Under silicon limitation, diatoms stop growing rapidly, whereas under nitrogen limitation, cells first consume intracellular nitrogen reserves and then stop growing. The consequence is that silicon starved cells maintain nitrogen reserves that enable a rapid recovery upon a pulse of nutrient while nitrogen starved cells are deficient in essential components (enzymes, chlorophyll) that are required for growth. In the natural environment, under conditions of nutrient limitation, diatoms are likely to become silicon limited when other phytoplankton species would be nitrogen or phosphorus limited. The diatoms would then be able to resume growth more quickly than the other species, which is consistent with their documented ability to outcompete other species during bloom events [75,76].

### Silicon transporter (SIT) gene clusters and identification of a new silicon transporter

**Figure 7:**
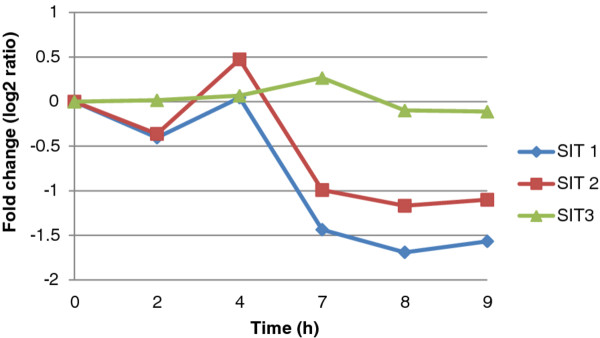
Expression pattern of SIT 1, 2 and 3.

**Figure 8:**
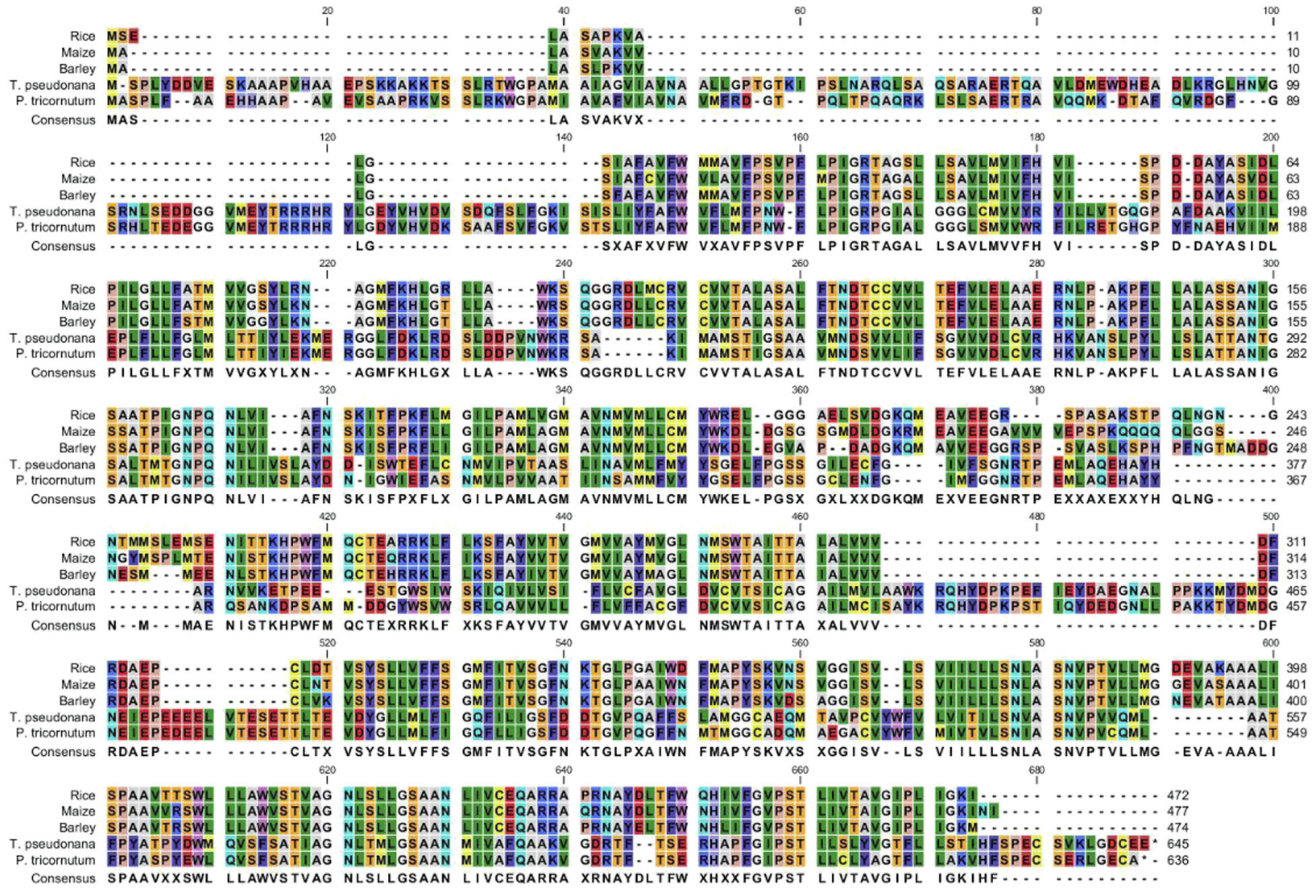
** Multiple alignment of Thaps3_21292 and plant silicon efflux transporters.** Sequence annotations: Rice,BAF73750.1; Maize, NP_001183945.1; Barley, BAH84976.1; *T. pseudonana, *Thaps3_21292; P. tricornutum, Phatr2_33218.

The expression patterns of the three SITs are shown in Figure 7. SIT1 and 2 are similarly regulated while SIT3 is different and does not substantially change (Figure 7). A total of 24 genes (Table 1) were identified that clustered with SIT1 (9 genes) and SIT2 (15 genes). The most interesting SIT1 co-regulated gene was Thaps3_21968, which encoded an unknown protein containing a domain that was found only in the diatom genomes. The domain consists of approximately 80 amino acids after accounting for insertions in individual sequences ( Additional file 12). *T. pseudonana* encoded 30 proteins with this domain, and *P. tricornutum* encoded 24. Of the 30 *T. pseudonana* genes, three (1891_bd, 24565, 9257) contained a multi-sensor signal transduction multi-kinase domain (COG3899), but the rest had no homologs. All of the proteins contained predicted coiled-coil domains, suggesting that this novel diatom-specific domain may play important roles in cellular processes involving protein-protein interactions. Thaps3_21968 was not only co-regulated with SIT1 during the synchrony, but during silicon starvation (data not shown), suggesting the possibility of a SIT-related function. The other genes exhibited no co-regulation to SIT1 or to each other. Of the SIT2 co-regulated genes, the most interesting was Thaps3_21292, which encoded what was annotated as a citrate transporter, but which upon BLAST analysis, proved to be a homolog to the silicon efflux transporter Lsi2 identified in plants with similarity ranging from E-value of 5e-31 to 7e-31 [77,78] (Figure 8). Thaps3_21292 had a single homolog in each of the other diatom genomes. It was also co-expressed with SIT2 during silicon starvation (data not shown). Interpro and NCBI CDD and PFAM analysis of Thaps3_21292 showed that it has arsenite (GO:0015105) and citrate transmembrane transporter activity (GO:0015137), and characterization of rice Lsi2 showed its involvement in arsenite efflux - thus this class of protein can also serve as a divalent anion symporter. The sequence similarity to well characterized silicon effluxers [77] and co-expression with the SITs strongly suggest that this gene encodes a new class of silicon efflux protein in diatoms. 

**Table 1:** Genes clustering with SITs

	**SIT 1 Cluster**
Thaps3_ protein ID	Protein Characteristics
9632	Hypothetical protein, serine rich, homolog to 10796
10796	Hypothetical protein, serine rich, homolog to 9632
21968	Diatom-specific protein with conserved domain
25551	Hypothetical protein
263046	Helicase, putative
263454	Trypsin-like serine protease
264646	Inverting glycoside hydrolase with beta-1,4-glucanase activity
268895	SIT 1, silicon transporter
1802_bd	Hypothetical protein
739_bd	Signal recognition particle receptor subunit alpha
	SIT2 Cluster
Thaps3_ protein ID	Protein Characteristics
41392	SIT2, Silicon transporter
21087	Hypothetical protein (123 aa) SP present, co-regulated with SIT2 under Si-
11091	Serine Threonine Protein kinase catalytic domain
5510	Glycosyltransferase family 10, alpha 1,3 fucosyltransferase
3131	Probable serine/threonine-protein kinase
24636	Hypothetical protein
23856	Fanconi anemia group D2 protein homolog, involved in homology-directed DNA repair
268582	Probable DNA helicase
38514	Multiple TM domains, two domains of unknown function (DUF3593 and DUF2499)
21292	Citrate transporter
263984	SprT metalloprotease domain
11282	Mannosyltransferase domain
21856	Hypothetical protein
7999	Hypothetical protein
8280	Hypothetical protein
4756	FlgN and Tat binding interacting protein domain

## Conclusions

The three datasets analyzed in this study have provided new insights into diverse aspects of silicon-related processes in diatoms, and further characterization of these genes could increase our understanding of biological interactions with silicon. The SLRG dataset contains a diverse set of genes that may be involved in other essential steps in the process of cell wall synthesis. Thus far only cell surface proteins and proteins tightly associated with silica involved in diatom cell wall formation have been characterized, and our current understanding of the molecular details of frustule formation is mostly static. The inability to isolate the SDV in pure form has prevented characterization of components associated with the SDV. In the SLRG dataset, in addition to identifying new genes potentially involved in silicification, we identified other genes potentially involved in other aspects of cell wall synthesis such as signaling, trafficking, protein degradation, glycosylation and transport. A large number of genes encoded yet unknown proteins and potential components of the cell wall formation machinery. Work is in progress to characterize the role of these proteins, including using localization with GFP fusions and knockdowns. The SSRG dataset represents the first documentation of a diatom’s genome-wide response to silicon starvation. The major finding from the SSRG dataset is that the overrepresentation of transcription and translation related genes indicates that *T. pseudonana* is poised to rapidly recover from silicon starvation and resume cell cycle progression upon silicon replenishment. This provides the first molecular data that explains the well-established environmental response of diatoms to grow as blooms and to out-compete other classes of microalgae for growth [75,76]. Further characterization of genes up-regulated under silicon starvation should provide insights into the mechanisms of silicon dependent cell cycle arrest and silicic acid sensing and signal transduction in diatoms. An important discovery, based on comparison with cell cycle arrest triggered by different conditions in a different diatom species, is that assignment of the cell cycle specific stage of various cyclins and cyclin dependent kinases should be re-evaluated under more diverse conditions and with other species. Finally, genes co-varying in expression pattern with the silicon transporters enabled identification of a new class of diatom-specific proteins containing a unique domain, and a putative diatom silicon effluxer. Thus far, the only models for the molecular recognition of silicon by proteins have been postulated for the SITs [79,80] these new proteins may provide more examples to evaluate.

## Methods

### Culture conditions and evaluation of the extent of synchrony

Axenic cultures of *Thalassiosira pseudonana* (CCMP1335) were synchronized as described [5]. Exponentially-grown cells were harvested by centrifugation, rinsed once and placed in silicate free medium and after 24 h, rhodamine 123 (R123) and 200 μM sodium silicate were added to the culture. Prior to and then every hour after silicate addition, 750 ml was removed, treated with cycloheximide (20 μg/ml), and harvested. Cells were pelleted and stored at −80°C prior to total RNA isolation [81]. To establish the validity of the synchrony, semiquantitative PCR was performed on silaffin 3 (Thaps3_25921) and compared with R123 incorporation ( Additional file 1: Figure S1). To provide a biological replicate and confirm the pattern of expression of genes determined by the Affymetrix analysis, quantitative real time PCR (qRT-PCR) was performed on several genes ( Additional file 1: Figure S2).

### Affymetrix array design, hybridization, and analysis

An Affymetrix GeneChip whole genome tiling array was designed, and analyzed at the gene and exon level analysis with a total of 524,909 sense strand probes (average of 16 probes per gene), based on gene model predicted transcripts for *T. pseudonana*, version 3.0 ( http://genome.jgi-psf.org/Thaps3/Thaps3.home.html). Microarrays were chosen as the platform for whole transcriptome analysis because they were the only method available to us to accommodate a statistically relevant number of conditions in a cost-effective manner. Considering the generally significant agreement between the two approaches, we expect that array data will continue to be useful for generation of experimentally robust and enduring insights [82]. The data discussed in this publication have been deposited in NCBI's Gene Expression Omnibus and are accessible through GEO Series accession number GSE37081 ( http://www.ncbi.nlm.nih.gov/geo/query/acc.cgi?acc= GSE37081). Included on the array were 33,886 antigenomic probes to account for nonspecific hybridization. For each hybridization, double stranded cDNA was synthesized from 7 μg of total RNA with no amplification using the GeneChip^®^ WT Amplified Double-Stranded cDNA Synthesis Kit (Affymetrix). Cleanup of double-stranded cDNA was done with the GeneChip^®^ Sample Cleanup Module (Affymetrix). Fragmentation and end-labeling was performed using the GeneChip^®^ WT Double-stranded DNA Terminal Labeling Kit (Affymetrix). Hybridization of labeled targets on the arrays was carried out using the GeneChip^®^ Hybridization, Wash, and Stain Kit (Affymetrix). The arrays were then scanned with the GeneChip^®^ Scanner, to generate the probe cell intensity data files. Initial data analysis, hierarchical clustering, and Blast2GO analyses were performed as described in Additional file 13.

### Characterization of the cell cycle and silaffin (Tpsil3) expression profile

The degree of synchronization of *T. pseudonana* was monitored by visualizing incorporation of rhodamine 123 (R123) into cell wall components and by measuring the mRNA expression pattern of Tpsil3 [Thaps3_25921 - [9]. This gene shows a pattern [9] of transient up-regulation during a portion of G1 (0–3 h after silicon addition) when girdle bands are synthesized, down-regulation during S-phase (3–4 h), up-regulation during valve formation (5–8 h) followed by down-regulation after cell separation (7–9 h). The precise timing of these events can vary in different synchronies [5], however, the Tpsil3 expression pattern has been established as a marker that enables correlation between different experiments [9]. For whole genome transcript profiling experiments reported here, both R123 incorporation and semi quantitative PCR on Tpsil3 were consistent with maximum valve formation occurring between 7–8 hours ( Additional file 1: Figure S1). A biological replicate was obtained on a separate synchrony by analyzing R123 incorporation and Tpsil3 mRNA levels using qRT-PCR. The data ( Additional file 1: Figure S1) confirmed synchronization after accounting for a shift in the timing of cell cycle events. Comparison of the subsequently-obtained Affymetrix data with 12 reference genes monitored by qRT-PCR ( Additional file 1: Figure S2) showed correlation between the datasets.

## Abbreviations

SDV: Silica deposition vesicle; SIT: Silicon transporter; SLRG: Silaffin-like response genes; SSRG: Silicon starvation responsive genes.

## Competing interests

The authors declare that they have no competing interests.

## Authors’ contributions

RPS, BT, MH produced biological samples, analyzed data and composed the paper. TNK and SF extracted and analyzed the initial microarray data. MH directed the biological aspects of the project, including data analysis. AE designed the microarrays and coordinated the microarray aspect of the project. All authors read and approved the final manuscript.

## Supplementary Material

Additional file 1 Figures S1 and S2. Click here for file

Additional file 2 ** Table S1.** 485 SLRG dataset. Click here for file

Additional file 3 ** Table S2.** SSRG dataset. Click here for file

Additional file 4 ** Figure S3.** GoSlim. Click here for file

Additional file 5 ** Figure S4.** Venn diagram of SLRGs. Click here for file

Additional file 6 ** Figure S5.** Choline transporter. Click here for file

Additional file 7 ** Table S3.** Mock vs SLRG dataset. Click here for file

Additional file 8 ** Table S4.** Characteristcs of silaffin cluster. Click here for file

Additional file 9 ** Figure S6.** SSRG cluster. Click here for file

Additional file 10 ** Figure S7.** Venn diagram of SSRG. Click here for file

Additional file 11 ** Table S5.** Mock Sapriel vs SSRG dataset. Click here for file

Additional file 12 ** Figure S8.** 21968 Domain alignment. Click here for file

Additional file 13 ** Method.** Initial data analysis. Click here for file
