# Supplementary material for: Whole transcriptome analysis of the silicon response of the diatom Thalassiosira pseudonana
Source: BMC Genomics. 2012 Sep 20;13:499. doi: 10.1186/1471-2164-13-499 (PMC3478156; doi:10.1186/1471-2164-13-499)
Supplement: Additional file 1 — Figures S1 and S2. [file 1471-2164-13-499-S1.pdf]

Additional File 1

Methods

Semi quantitative PCR

Total RNA extracted [1] with Trizol reagent (Sigma) from cells harvested at each time point was treated with RNAse-free DNAse followed by purification and concentration using RNeasy Plus mini kit (Qiagen). cDNA was synthesized from equal amounts of purified total RNA using Superscript III reverse transcriptase (Invitrogen). The silaffin 3 (Thaps3_25921) gene was amplified using sense primer R-154-for (5'-GTTGAGGAGGATGTTGCTGGC-3') and antisense primer R-155-rev (5'-TCAAGCGCTCATGGAGTGGAC-3'). A standard PCR program of initial denaturation at 95°C (2 m) followed by 15, 20, 25 and 30 cycles of denaturation (95°C, 30 s), annealing (60°C, 30 s) and extension steps (72°C, 30 s) was used. Densitometric analysis of the electronic gel image was performed using UN-SCAN-IT v6.1 software (Silk Scientific, UT).

Quantitative Real time PCR (qRT-PCR)

Quantitative real time PCR was performed with equal amounts of cDNA generated as described above, using a Light Cycler 2.0 and the Fast Start DNA Master^{Plus} Sybr Green I kit (Roche Applied Science). Primers for qRT PCR (Table SI 1) were designed using the IDT's real-time primers design tool (http://www.idtdna.com/scitools/Applications/RealTimePCR/). Standards for real time PCR were dilutions of *T. pseudonana* genomic DNA. Normalization was done using TATA-box binding protein (Thaps3_264095) as a house keeping gene [2]. Dilutions of cDNA were done to ensure that amplifications were in the linear range.

Results

Initial determination of the extent of cell cycle synchronization was done using semi-quantitative PCR on TpSil3 using RNA extracted every hour after silicon re-addition (Figure S1A). A biological replicate synchrony was done to generate cDNA for qRT-PCR analysis for TpSil3 (Figure S1B) and other genes listed in Table S1. After microarray data processing, the pattern for TpSil3 is displayed in Figure S1C. There is a shift in timing in the biological replicate (Figure S1B) relative to the microarray synchrony (Figure S1C), which and has been previously documented [1] and is not unusual.

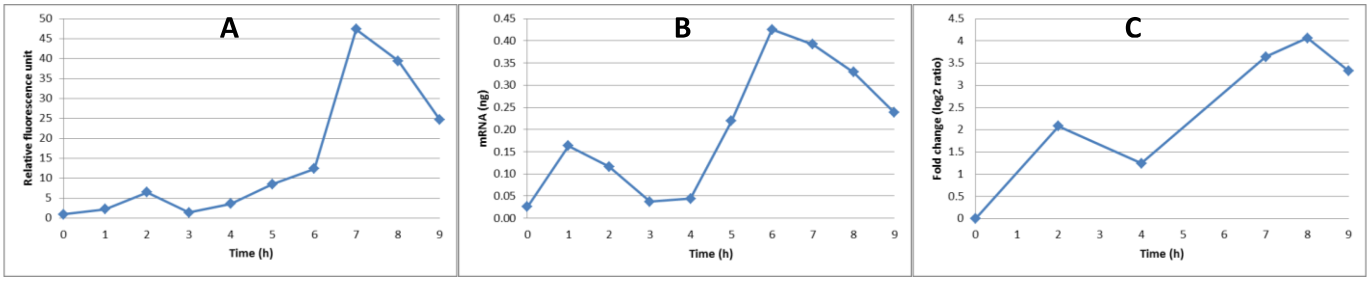

Figure S1. Tpsil3 (Thaps3_25921) expression profiles. A, semi-quantitative PCR of the samples harvested at each hour of the cell cycle. B, qRT-PCR of a biological replicate synchrony. C, The microarray expression profile of the same gene.

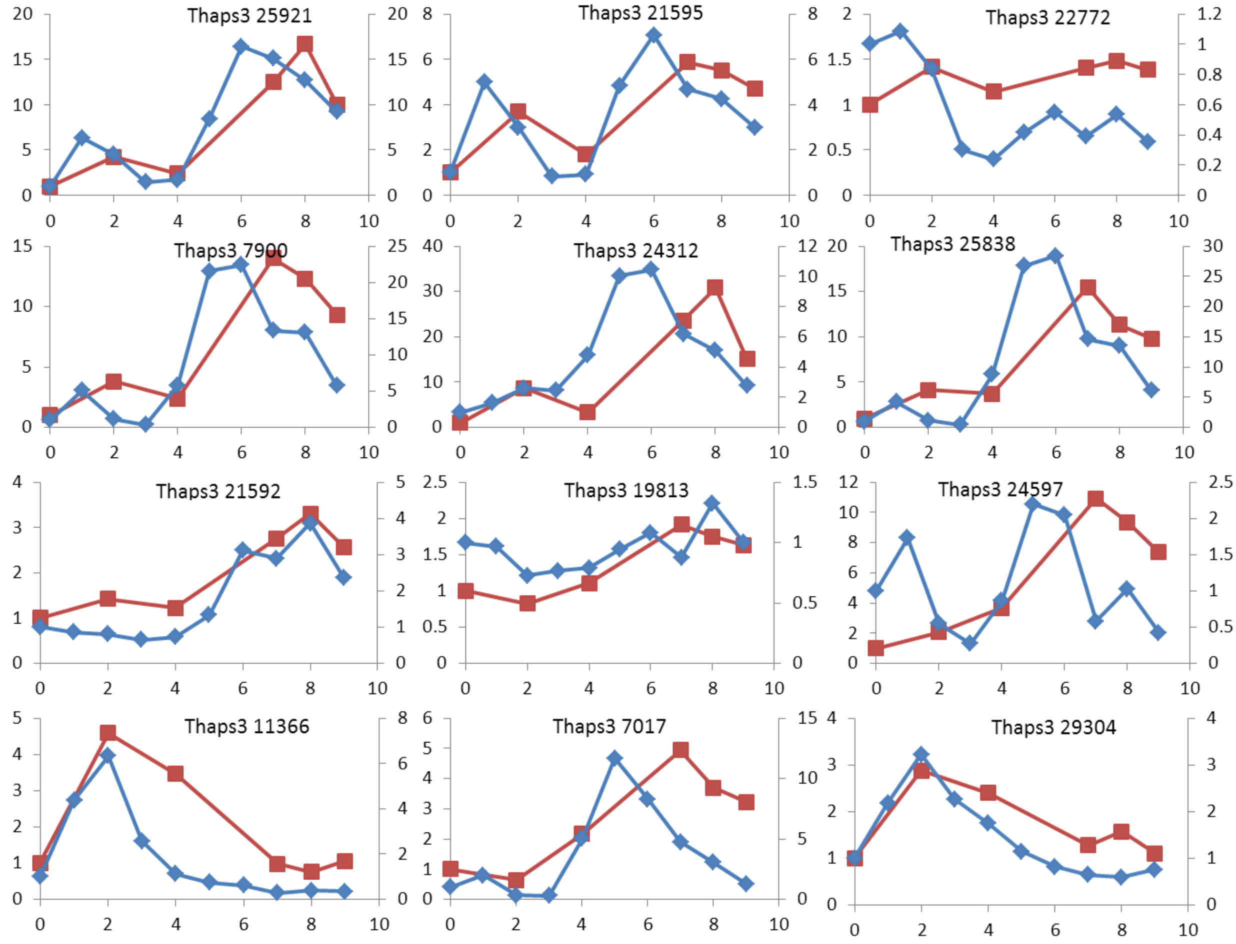

Figure S2. Verification of microarray data (red) by real-time quantitative PCR (blue) performed with corresponding primer pairs listed in SI material and methods section. Y-axis represents mRNA in relative amount for microarray on the left and qPCR on the right.

1. Hildebrand M, Frigeri L, Davis AK: **Synchronized growth of *Thalassiosira pseudonana* (Bacillariophyceae) provides novel insights into cell wall synthesis processes in relation to the cell cycle.** *J Phycol* 2007, **43:**730-740.
