# Supplementary material for: Whole transcriptome analysis of the silicon response of the diatom Thalassiosira pseudonana
Source: BMC Genomics. 2012 Sep 20;13:499. doi: 10.1186/1471-2164-13-499 (PMC3478156; doi:10.1186/1471-2164-13-499)
Supplement: Additional file 4 — Figure S3. GoSlim. [file 1471-2164-13-499-S4.pdf]

Additional File 4

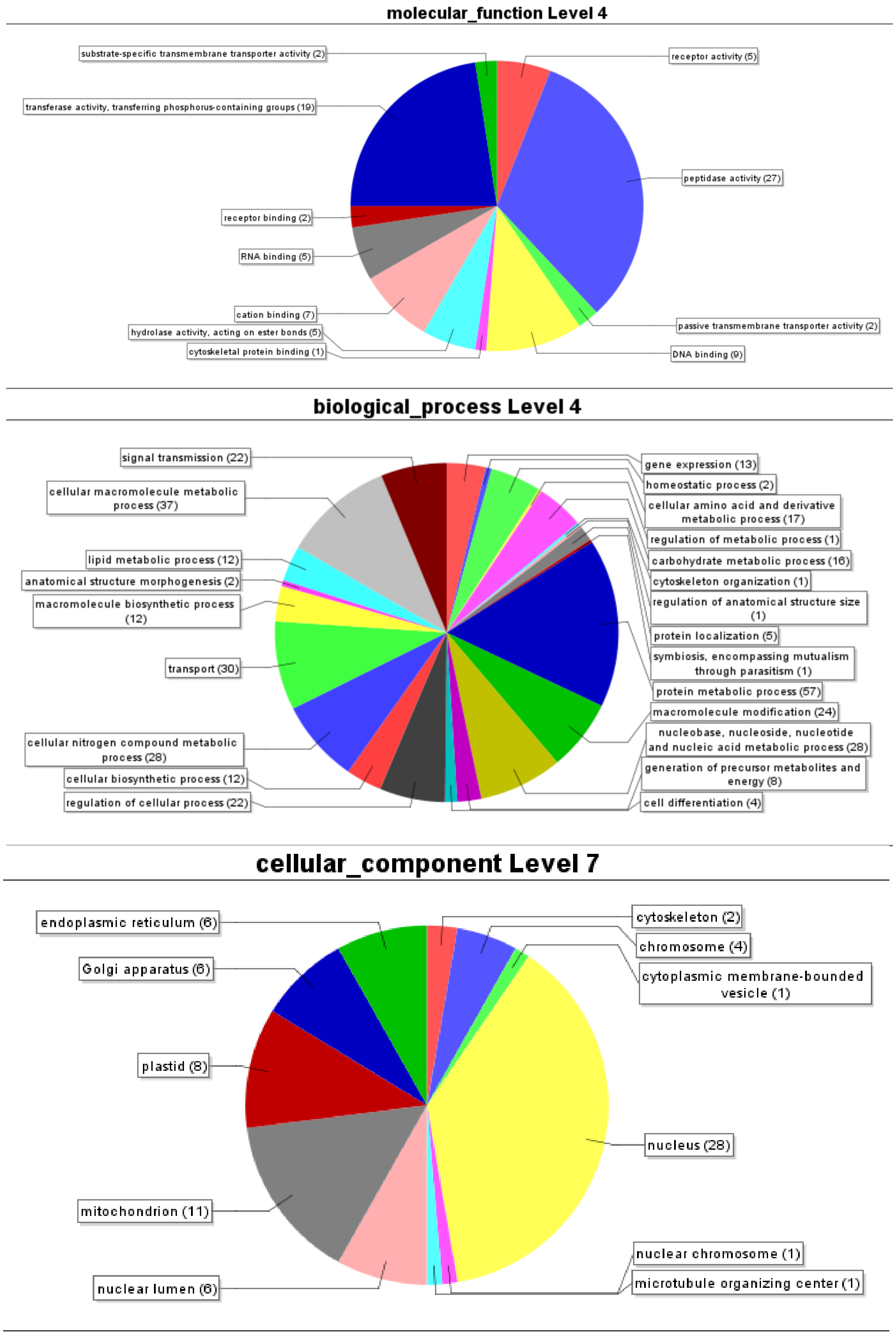

Figure S3. GO slim functional characterization of SLRGs. Pie diagrams showing Molecular function (249 genes), Biological Process (181 genes) and Cellular components level (125 genes).

The pie chart of molecular functions at level 4 indicated that the genes with peptidase activity (27 genes) constituted the largest component, followed by transferase activity (transfer of phosphate containing group) (19 genes). Similarly, the pie chart of biological process at level 4 showed protein metabolic process contained the highest number of genes (57 genes) followed by transport (30 genes). Cellular component at level 7 shows most genes are components of the nucleus (28), followed by mitochondria (11 genes), ER (6 genes), Golgi (6 genes) and plastid (6 genes).
