# Supplementary material for: Whole transcriptome analysis of the silicon response of the diatom Thalassiosira pseudonana
Source: BMC Genomics. 2012 Sep 20;13:499. doi: 10.1186/1471-2164-13-499 (PMC3478156; doi:10.1186/1471-2164-13-499)
Supplement: Additional file 5 — Figure S4. Venn diagram of SLRGs. [file 1471-2164-13-499-S5.pdf]

Additional File 4

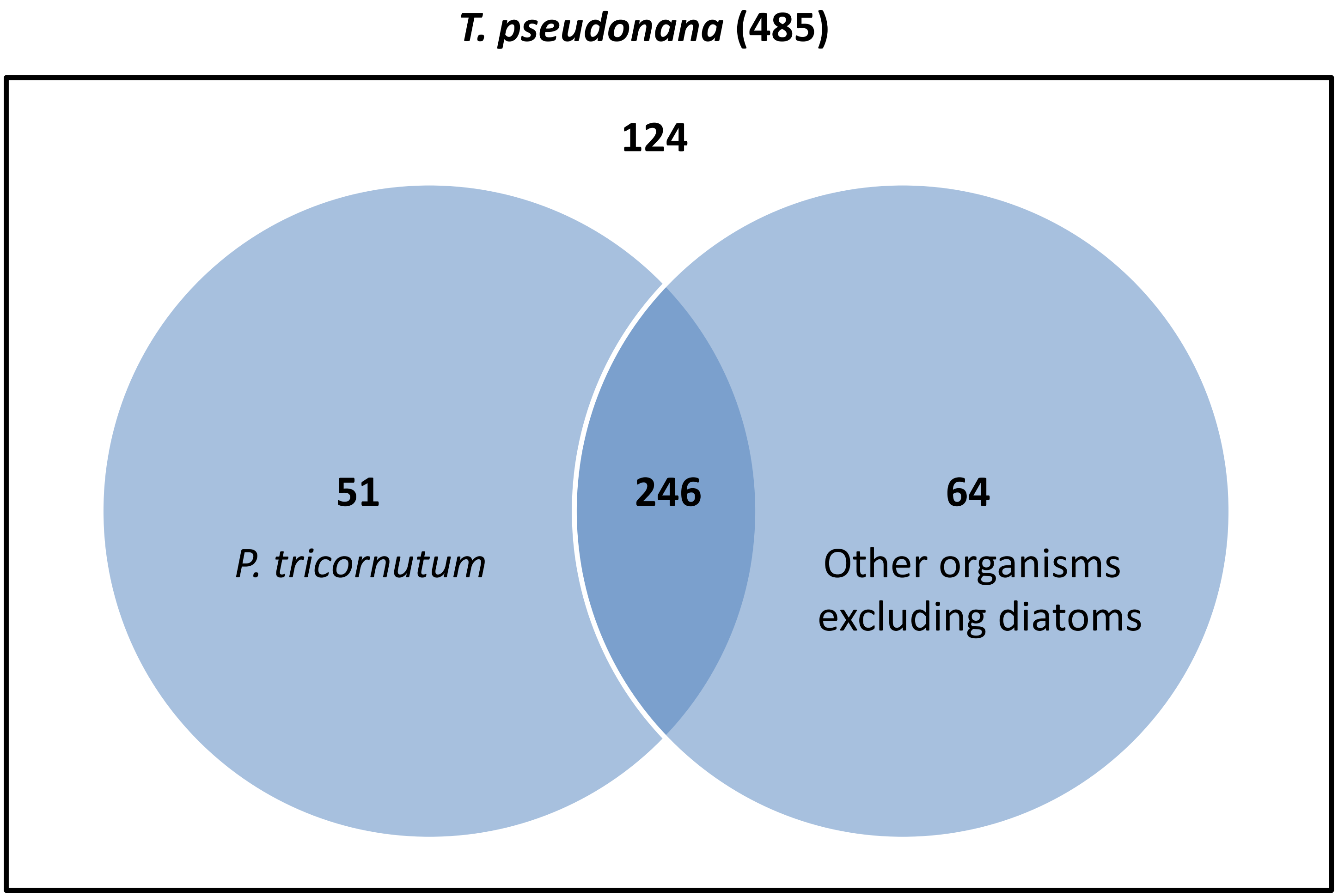

Figure S4. Venn diagram of 485 genes (SLRG) showing 175 genes were specific to diatoms, among which 124 genes were so far found only in *T. pseudonana*. Based on NCBI blast analysis. E-value cutoff 1E-10.
