# Supplementary material for: Whole transcriptome analysis of the silicon response of the diatom Thalassiosira pseudonana
Source: BMC Genomics. 2012 Sep 20;13:499. doi: 10.1186/1471-2164-13-499 (PMC3478156; doi:10.1186/1471-2164-13-499)
Supplement: Additional file 6 — Figure S5. Choline transporter. [file 1471-2164-13-499-S6.pdf]

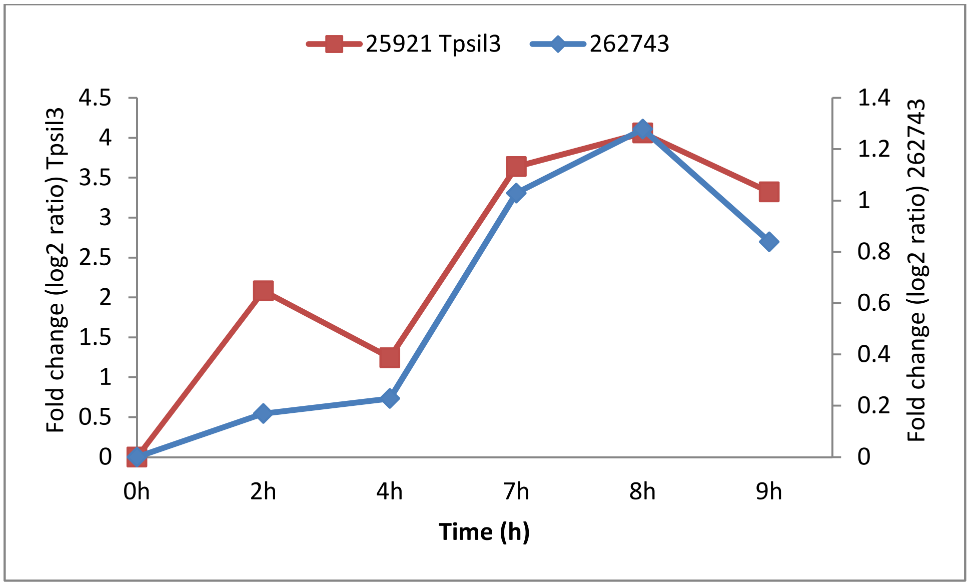

Additional File 5

Figure S5 Choline transporter-like protein Thaps3_262743 and Tpsil3 have similar expression pattern during silica replete cell cycle.
