# Supplementary material for: Whole transcriptome analysis of the silicon response of the diatom Thalassiosira pseudonana
Source: BMC Genomics. 2012 Sep 20;13:499. doi: 10.1186/1471-2164-13-499 (PMC3478156; doi:10.1186/1471-2164-13-499)
Supplement: Additional file 9 — Figure S6. SSRG cluster. [file 1471-2164-13-499-S9.pdf]

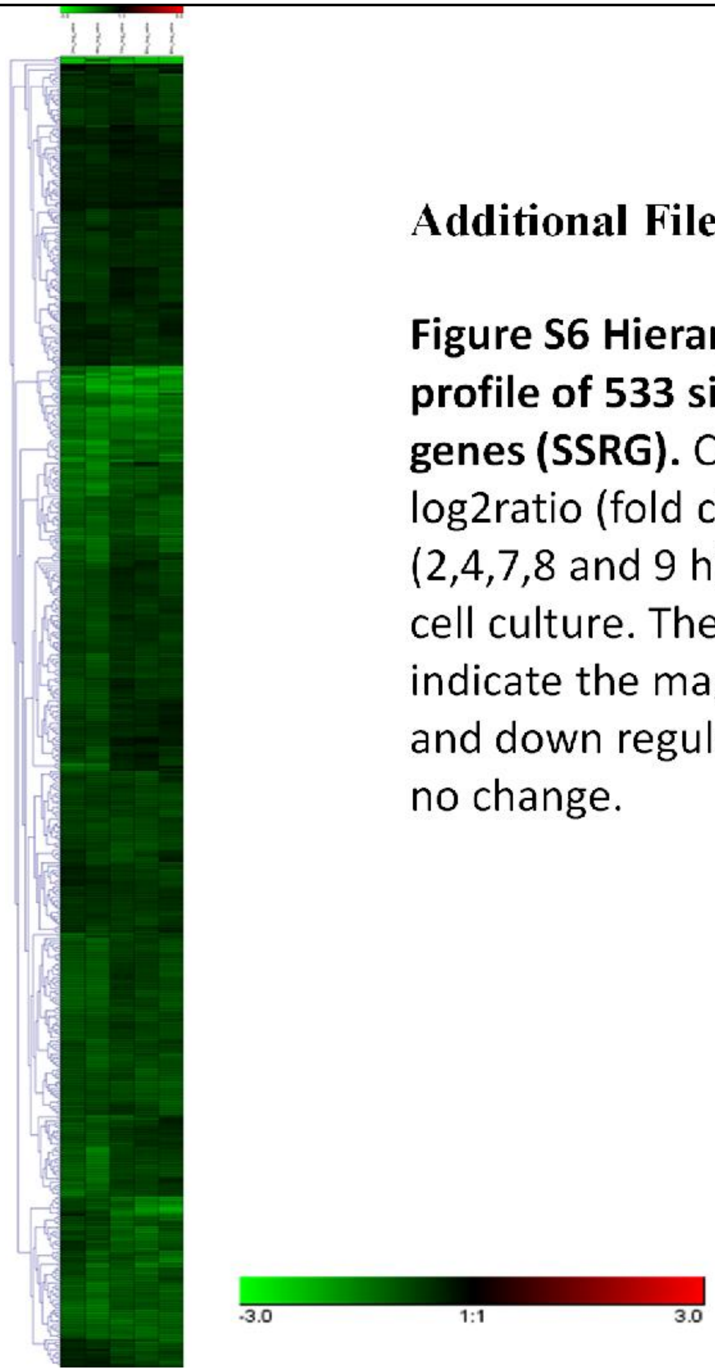

Additional File 8

Figure S6 Hierarchical clustered expression profile of 533 silicon starvation responsive genes (SSRG). Columns correspond to log2ratio (fold change) of the time points (2,4,7,8 and 9 h) relative to 0h of synchronized cell culture. The intensities of the colors indicate the magnitude of upregulation (red) and down regulation (green). Black indicates no change.
