# Supplementary material for: Whole transcriptome analysis of the silicon response of the diatom Thalassiosira pseudonana
Source: BMC Genomics. 2012 Sep 20;13:499. doi: 10.1186/1471-2164-13-499 (PMC3478156; doi:10.1186/1471-2164-13-499)
Supplement: Additional file 10 — Figure S7. Venn diagram of SSRG. [file 1471-2164-13-499-S10.pdf]

Additional File 9

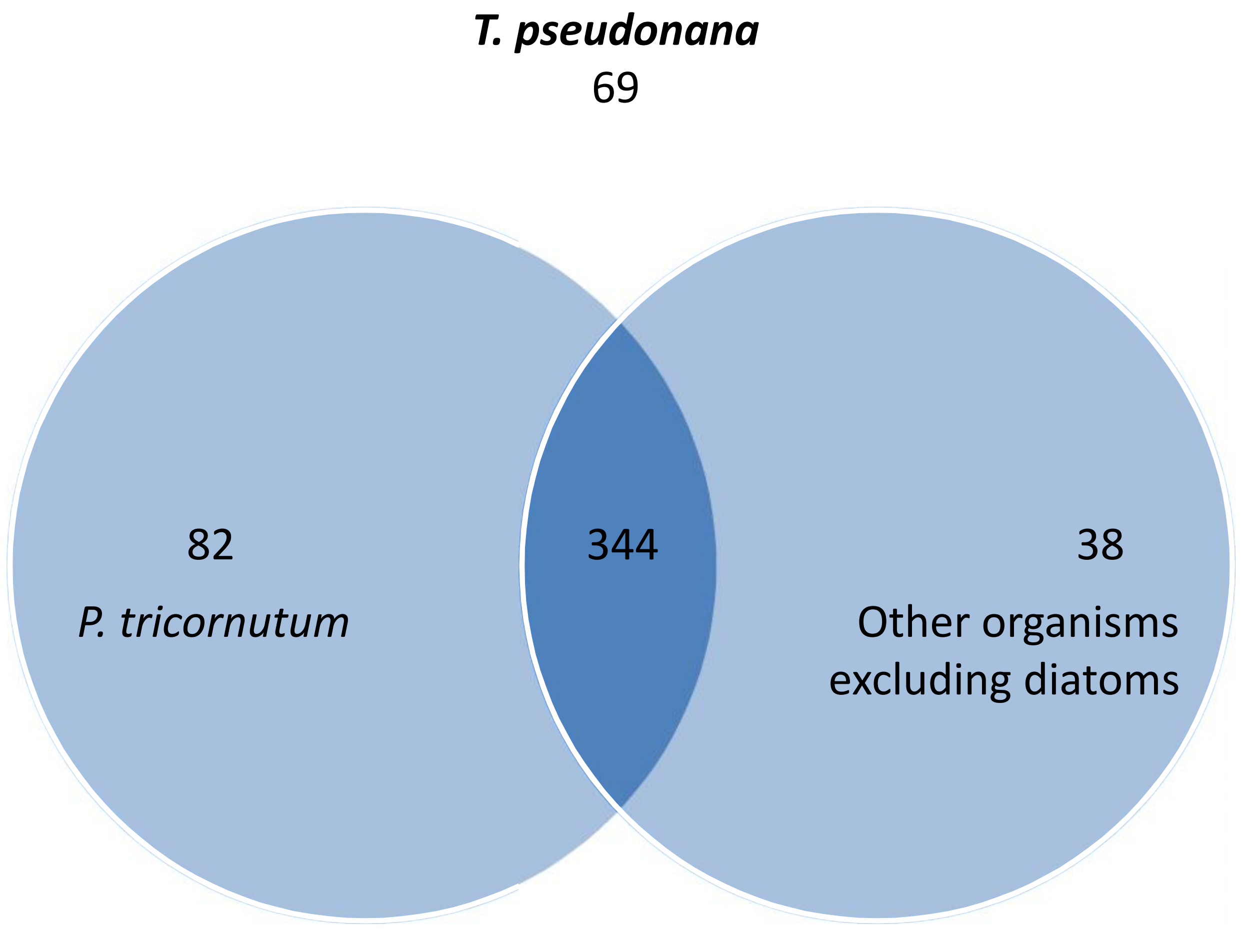

Figure S7. Venn diagram of 533 genes (SSRG) upregulated at 0 h showing 151 genes were specific to diatoms, among which 69 genes were so far found only in *T. pseudonana*. Based on NCBI blast analysis. E-value cutoff 1E-10.
