# Supplementary figures and images for: Whole transcriptome analysis of the silicon response of the diatom Thalassiosira pseudonana
Source: BMC Genomics. 2012 Sep 20;13:499. doi: 10.1186/1471-2164-13-499 (PMC3478156; doi:10.1186/1471-2164-13-499)

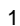

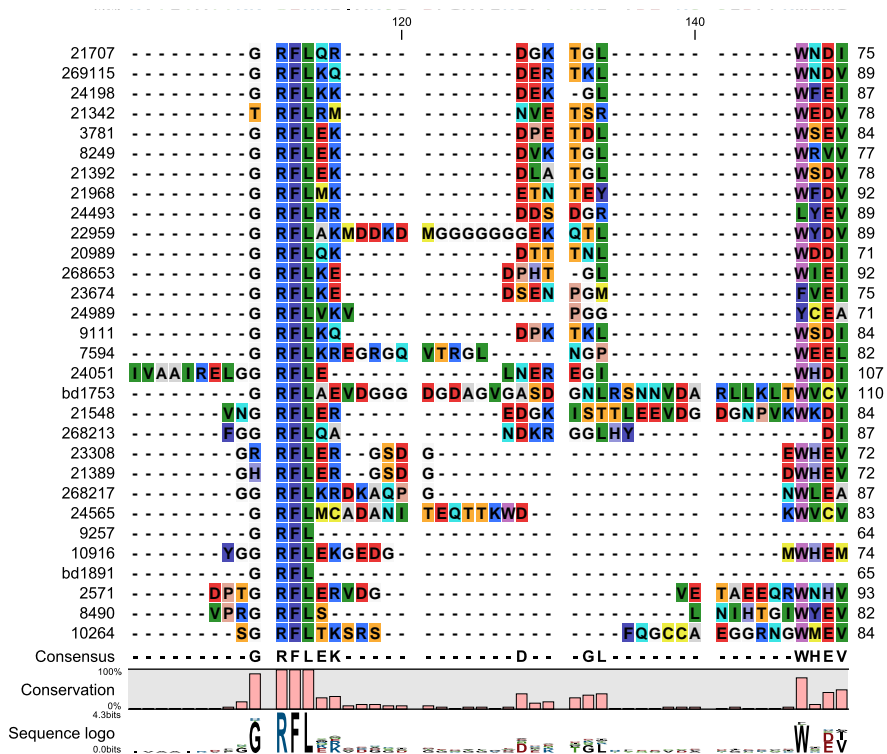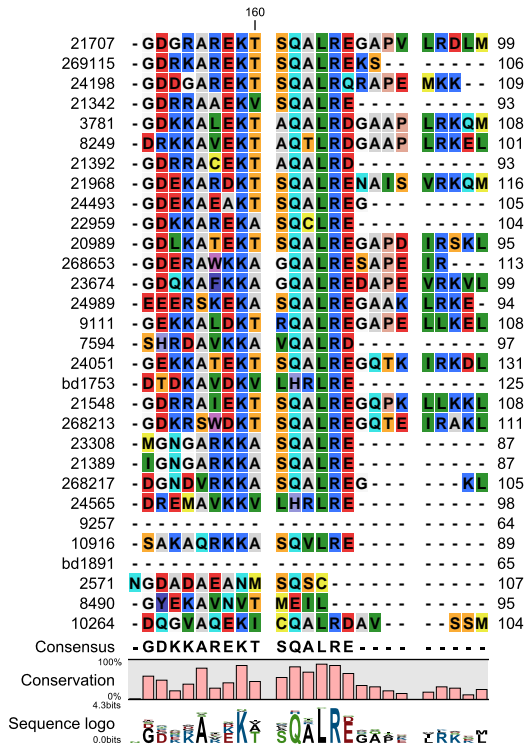

Supplement: Additional file 12 — Figure S8. 21968 Domain alignment. [file 1471-2164-13-499-S12.pdf]
