# Supplementary material for: Whole transcriptome analysis of the silicon response of the diatom Thalassiosira pseudonana
Source: BMC Genomics. 2012 Sep 20;13:499. doi: 10.1186/1471-2164-13-499 (PMC3478156; doi:10.1186/1471-2164-13-499)
Supplement: Additional file 13 — Method. Initial data analysis. [file 1471-2164-13-499-S13.pdf]

## Additional File 13

### Affymetrix initial data analysis

Initial analysis of gene level expression was performed using Affymetrix Expression Console Software, version 1.1. The Robust Multichip Analysis (RMA) algorithm was applied to the probe cell intensity data files for all experimental conditions, using default parameters in the RMA-sketch workflow for core gene level analysis. Hybridization results were visually inspected for outliers using the Affymetrix Expression Console quality control metrics. Log base two normalized intensities were then exported based on probe set identifiers. The probe set identifiers in the summarization files were mapped to the filtered gene model predicted transcript identifiers for *T. pseudonana*, version 3.0. Gene annotation mapping for the transcripts was obtained from a database created and maintained at the J. Craig Venter Institute. The annotations were appended to the intensity summarization files and converted to tab-delimited text files.

Normalized and mapped probe intensities were then subjected to a two-pronged test for differential expression which included a false discovery rate (FDR) corrected student t-test statistic along with a log fold change cutoff of +/- 1. Two tailed unpaired t-tests were performed between time-points using the standard function within Microsoft Excel. The FDR correction was performed via the method of Benjamini and Hochberg [3] implemented by the R statistical software package. Fold changes were calculated as the difference of any time-point from the initial 0hr time-point. Genes were considered to be differentially expressed if they had an FDR corrected p-value $< .05$ and a fold change greater or less than +/- 1.

### Hierarchical clustering

Standard hierarchical clustering was carried out with Genesis v1.7.6 [4] using default settings of an average linking clustering. We only clustered genes which fell below the differential expression cutoffs described above for at least one sample point in the time course.
